# Versatile Nasal Application of Cyclodextrins: Excipients and/or Actives?

**DOI:** 10.3390/pharmaceutics13081180

**Published:** 2021-07-30

**Authors:** Giovanna Rassu, Milena Sorrenti, Laura Catenacci, Barbara Pavan, Luca Ferraro, Elisabetta Gavini, Maria Cristina Bonferoni, Paolo Giunchedi, Alessandro Dalpiaz

**Affiliations:** 1 Department of Chemistry and Pharmacy, University of Sassari, Via Muroni 23a, I-07100 Sassari, Italy; grassu@uniss.it (G.R.); eligav@uniss.it (E.G.); 2 Department of Drug Sciences, University of Pavia, Viale Taramelli 12, I-27100 Pavia, Italy; milena.sorrenti@unipv.it (M.S.); laura.catenacci@unipv.it (L.C.); cbonferoni@unipv.it (M.C.B.); 3 Department of Neuroscience and Rehabilitation—Section of Physiology, University of Ferrara, Via Borsari 46, I-44121 Ferrara, Italy; pvnbbr@unife.it; 4 Department of Life Sciences and Biotechnology, University of Ferrara, Via Borsari 46, I-44121 Ferrara, Italy; frl@unife.it; 5 Department of Chemical, Pharmaceutical and Agricultural Sciences, University of Ferrara, Via Fossato di Mortara 19, I-44121 Ferrara, Italy; dla@unife.it

**Keywords:** cyclodextrin, intranasal, formulation, local therapy, systemic drug delivery, nose-to-brain

## Abstract

Cyclodextrins (CDs) are oligosaccharides widely used in the pharmaceutical field. In this review, a detailed examination of the literature of the last two decades has been made to understand the role of CDs in nasal drug delivery systems. In nasal formulations, CDs are used as pharmaceutical excipients, as solubilizers and absorption promoters, and as active ingredients due to their several biological activities (antiviral, antiparasitic, anti-atherosclerotic, and neuroprotective). The use of CDs in nasal formulations allowed obtaining versatile drug delivery systems intended for local and systemic effects, as well as for nose-to-brain transport of drugs. In vitro and in vivo models currently employed are suitable to analyze the effects of CDs in nasal formulations. Therefore, CDs are versatile pharmaceutical materials, and due to the continual synthesis of new CDs derivatives, the research on the new nasal applications is an interesting field evolving in the coming years, to which Italian research will still contribute.

## 1. Introduction

Nasal drug delivery can be a good alternative route for the administration of drugs intended for systemic and brain targeting, particularly for drugs with low oral bioavailability due to high hepatic first-pass metabolism and/or a low permeation through the Blood-Brain Barrier (BBB). Cyclodextrins (CDs) can be useful as pharmaceutical excipients for their capability to act as solubilizers and absorption promoters in nasal drug delivery [1].

CDs’ history began in the late 19th century with their discovery in 1891 by Antoine Villiers; Franz Schardinger, discovering in 1903 that a microorganism was responsible for starch degradation forming crystalline products very similar to the two cellulosine α-dextrin and β-dextrin, described by Villiers, is still now considered the father of CDs because he was the first researcher that described their properties, including their complexation ability [2].

**Figure 1:**
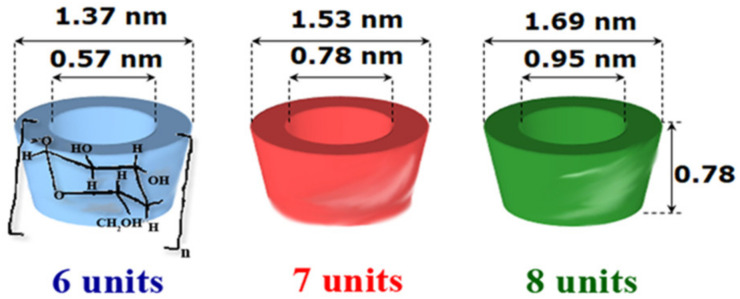
Structure and shape of native CDs.

CDs are cyclic oligosaccharides consisting of at least six (α-1,4)-linked D-glucopyranose units [3]. Native CDs used are α-CD, β-CD and γ-CD, consisting of six, seven, and eight glucopyranose units, respectively (Figure 1). If steric hindrances prevent the formation of CDs with less than six glucopyranose units, CDs with more than eight glycosidic units, commonly referred to as large-ring CDs (δ-CD, ε-CD, ι-CD, and η-CD), are difficult to produce and are not suitable to form inclusion complexes [4]. CDs have a typical truncated cone shape, in which the secondary hydroxyl groups (C_2_ and C_3_ carbon atoms of the glucose units) are located at the wider end of the cavity, and the primary hydroxyl groups (C_6_ carbon atoms of the glucose units) at the other narrower one (Figure 1). The presence of the hydroxyl groups on the edges of the truncated cone is responsible for the hydrophilic character of the CDs and their solubility in the aqueous medium. The presence of hydrogen atoms and glycosidic oxygen bridges in the internal cavity of CDs are responsible for the lipophilic character, enabling them to form inclusion complexes with hydrophobic molecules of proper dimensions [5].

Although the literature is mainly focused on the ability of CDs to form inclusion complexes, in the last decade, studies concerning the non-inclusion complexes were also reported, in which self-assembled nanoaggregates are formed as drug—CD interaction products [6].

The differences between the native CDs, in addition to the molar mass, are the size of the cavity due to the different number of glucose units and the aqueous solubility. β-CD have a limited water solubility due to the formation of intramolecular hydrogen bonds between the C_2_-OH group of a glucopyranoside unit with the C_3_-OH group of the adjacent one, which reduces their interactions with the water molecules. Nevertheless, β-CD has the most suitably sized cavity for complexing different guest molecules, so it remains the CD more used in pharmaceutical formulations [7].

CD derivatives have been developed to improve the physicochemical characteristics and the complexing ability of native CDs. In particular, in β-CD derivatives, the introduction of substituent on hydroxyl groups such as hydroxypropyl (HP-β-CD), methyl (heptakis(2,6-di-O-methyl)-β-CD (DM-β-CD), and randomly methylated β-CD (RM-β-CD)) and sulfobutylether sodium salt (SBE-β-CD) improved its aqueous solubility and toxicological profile. On the other hand, hydrophobic CD derivatives such as triacetyl CDs can be used as excipients to sustain and control the release of water-soluble drugs [8,9,10].

CDs are widely used in many industrial products, technologies, and analytical methods in various sectors such as pharmaceuticals, cosmetics, food, and agriculture. In the pharmaceutical field, CDs can be employed as excipients in conventional dosage form [11], in drug delivery systems [12], and, in most recent years, two CDs are approved as the active ingredients: HP-β-CD as orphan drug designation in the treatment of Niemann-Pick type C disease and a modified anionic γ-CD derivative, Sugammadex/Bridion^®^, in clinical anesthesia [13].

As excipients, the most relevant use of CDs is as a complexing agent to improve drug solubility and bioavailability; the CD complexation can also be exploited to ameliorate the stability of drugs, in terms of reduction of the evaporation of volatile substances or protection of drugs from light and/or oxygen-induced degradation, as well as to cover bad flavors or scents or transform liquid substances into powders and, in general, to prevent incompatibility reactions between substances in the formulation [14].

Inclusion complexes are formed by molecular encapsulation of a drug into the hydrophobic cavity of the CD; this process, due to the formation of non-covalent bonds between the guest (drug) and host (CD), is described by the following reversible reaction showing that guest molecules are in equilibrium with free molecules in the solution:

(1) $$a\;D\;+\;b\;\mathrm{CD}\;⇄D_{a}{\mathrm{CD}}_{b}$$

where *a* drug molecules (D) react with *b* CD molecules to form a complex of *a:b* stoichiometry. K*_a:b_* is the stability constant of the complex that can be written as follows:

(2) $$K_{a:b}=\frac{\left[D_{a}{\mathrm{CD}}_{b}\right]}{\left[D_{a}\right]\left[{\mathrm{CD}}_{b}\right]}$$

where the brackets refer to the molar concentration, and therefore, the unit of measurement of K*_a:b_* is *M*^−1^ [15]. In the pharmaceutical field, the most common stoichiometry of an inclusion complex is equimolecular 1:1, although it is possible to form complexes in different molar ratios higher in drug and/or CD. A plot of drug concentration in solution versus CD concentration for the formation of a 1:1 complex should give an A_L_-type phase-solubility diagram; the y-intercept represents the intrinsic drug solubility (*S_0_*), and by the slope of the straight line it is possible to calculate the stability constant according to the equation described by Higuchi and Connors [16]:

(3) $$K_{1:1}=\frac{\mathrm{slope}}{S_{0}\;\left(1-\mathrm{slope}\right)}$$

The ability of CDs to form inclusion complexes can be used in drug formulation to improve the solubility of poorly water-soluble class II and some class IV BCS drugs [17].

The choice of a specific CD to develop a pharmaceutical dosage form depends on several factors, such as characteristics of solubility, compatibility with the host, the route of administration, and the cost. β-CD is the most used CD in pharmaceutical products, and it is of easy production and the least expensive between oligosaccharides. Because of their very low oral bioavailability, β-CD and the other parent CDs are not toxic after oral administration, as well as most of their derivatives. Instead of parenteral administration, α-CD and β-CD are not suitable, showing renal toxicity. γ-CD, although less toxic, is not used in Europe for intravenous administration because it tends to form visible aggregates in aqueous solutions. Concerning the derivatives CDs, methylated β-CDs have been reported renal toxicity; therefore, they are not used in parenteral formulation, but RM-β-CD is used in the topical or nasal formulation; HP-β-CD and SBE-β-CD instead have also been approved for parenteral use [18].

α-CD (alfadex), β-CD (betadex), and γ-CD (gammadex) are listed in the European Pharmacopoeia (Ph.Eur.), the United States Pharmacopeia/National Formulary (USP-NF) and the Japanese Pharmaceutical Codex (JPC). The derivatives HP-β-CD (hydroxypropyl-betadex) are referenced in a monograph in the Ph. Eur. and together with SBE-β-CD (betadex sulfobutyl ether sodium) in the USP-NF. All native CDs have been introduced in the GRAS (generally regarded as safe) FDA list for use as food additives. β-CD is approved in Europe as a food additive (E 459) with an ADI (acceptable daily intake) of 5 mg/kg/day; SBE-β-CD and HP-β-CD are cited in the FDA’s list of Inactive Pharmaceutical Ingredients.

According to the EMA, SBE-β-CD and HP-β-CD are compatible with nasal delivery and well tolerated by the nasal mucosa in solutions up to 10% (HP-β-CD). RM-β-CD can be used in solutions up to 10% too, and together with DM-β-CD, it can have a direct action on the mucosal membranes improving the nasal permeability. β-CD can be used in solutions at a maximum of 1.5%, due to its low aqueous solubility; on the other hand, it is used successfully in solid products as dry powder formulations [19].

More than 80 pharmaceutical products are on the worldwide market containing CDs, and of these, two products were approved by FDA and EMA in 2019 for nasal administration. Baqsimi^TM^ (Eli Lilly, Indianapolis, IN, USA) is a nasal dry powder formulation containing betadex as a permeation enhancer for glucagon, used to treat severe hypoglycemia in patients with diabetes. Instead, Aerodiol^®^ (Servier, Suresnes, France) is a nasal spray solution of 17β-estradiol hemihydrate containing randomly methylated betadex used for hormone replacement therapy [11].

In addition to the products currently on the market, it is worth mentioning the more recent investigations on nasal and intranasal formulations where the promising role of CDs is focused on their ability to increase drug solubility and permeability through nasal mucosa [20].

## 2. Cyclodextrin as Excipient in Nasal Formulations

Nasal formulations obtained in the presence of CDs have been proposed for local therapy, systemic absorption, or brain targeting of the active drugs.

The nasal cavity is divided into three regions: vestibular, respiratory, and olfactory. After nasal administration, the formulation components that are able to overcome the vestibular region can be deposed on the mucosa of the other two regions. Therefore, the active drugs can perform local effects, or they can reach the bloodstream or the central nervous system (CNS) if they are able to permeate across the mucosa of the respiratory or olfactory regions, respectively. Indeed, the respiratory region is highly vascularized with blood vessels, constituting the largest area of the nasal cavity; the olfactory region is instead located in the upper portion of the nasal cavity where entry into the central olfactory sensory neurons is allowed. The drugs permeating across the respiratory mucosa can easily reach the bloodstream, whereas those permeating the olfactory mucosa can directly target the cerebrospinal fluid (CSF) where it is located in the olfactory bulb [21,22]. In the respiratory and olfactory mucosae, a small portion of trigeminal neurons is also included [23]. As a consequence, the access of drugs in the CNS after nasal administration is generally allowed by one or more of these three main pathways: (i) in the respiratory region, the drug may be absorbed into the bloodstream from which it can target the brain by crossing the BBB (systemic pathway); (ii) in the olfactory region, the drug may directly target the CSF by permeation across the olfactory mucosa, or reach the olfactory bulb by transcellular transport via olfactory neurons (olfactory pathway); (iii) the drugs penetrating in the respiratory or olfactory mucosa can reach the portions of trigeminal neurons, and then they can be transported via trigeminal nerves (trigeminal pathway) [24]. The respiratory pathway is accessible only to the drugs able to cross the BBB; otherwise, this way permits the drug absorption anyhow in the bloodstream; the olfactory or trigeminal pathways induce the drugs to follow an extracellular route, allowing for their delivery from the nose to the CSF or brain parenchyma within few minutes without binding to any receptor or undergoing axonal transport [25]. However, it is possible that some drugs can be axonally transported into the brain after endocytosis, but this process requires up to 48 h [25,26].

### 2.1. Nasal Formulations and the Role of CDs as Modulators of the Drug Absorption

Nasal formulations are therefore required to provide the drug deposition on the mucosa of the nose with prolonged residence time. When the drug absorption is wanted, it is also necessary to produce high local drug concentration on the nasal mucosa and increase its permeation ability to produce efficient diffusion patterns toward the bloodstream or the CNS. In this regard, chitosan is a widely used biocompatible polymer which in nasal formulations shows mucoadhesive properties due to its positive charges interacting with negatively charged mucosal surfaces [27,28]. Moreover, chitosan appears to transiently increase the permeability of mucosa by opening their tight junctions [29]. Taking into account these aspects, nasal formulations based on micro or nanoparticulate systems able to produce high dissolution rates of neuroactive drugs or prodrugs were designed; in these formulations, the presence of chitosan appeared important in inducing their efficient delivery in the CNS [30,31,32,33,34,35,36,37,38,39,40,41].

About the nasal mucosa, an unstirred water layer (UWL) contributes to their overall drug permeation barrier, constituting a diffusion layer. This is a typical physiologic system where CDs can significantly enhance drug permeation through lipophilic membranes [3,42]. On the contrary, CDs are not able to increase the drug permeation when the UWL is absent from the physiologic membranes [42]. Several mechanisms have been proposed to elucidate the drug permeation increase across the mucosa induced by CDs. Firstly, to enhance the solubility in water of poorly soluble drugs, the CDs can increase the concentration gradient over the lipophilic membranes of drugs that are essential for their passive diffusion processes [3]. Moreover, it is hypothesized that poorly water-soluble drug/CD complexes can form nanoparticles that are able to reduce the interaction of lipophilic drugs with the mucus, enhancing their permeation across mucus layer and, therefore, allowing a direct deposition of the drugs on the lipophilic membranes upon dissociation from the CDs [42,43]. Again, it is known that the CDs can form complexes with natural hydrophobic molecules belonging to the membranes, according to host–guest interactions. In particular, α-CDs and β-CDs are able to form complexes with phospholipids and cholesterol, respectively, inducing an increase in membrane fluidity [43,44].

Recent studies suggest that the CDs can differently interact with the respiratory and olfactory mucosa due to their different cell composition [45]. In particular, four types of cells constitute the respiratory epithelium: basal cells, goblet cells, non-ciliated columnar cells, and ciliated columnar cells. The cilia’s movement is coordinated, allowing it to move the mucus towards the pharynx. The olfactory epithelium is constituted by Bowman’s glands, which produce and secrete mucus, basal cells, sustentacular cells, olfactory sensory neurons, and the trigeminal nerve. Therefore, this mucosa is not provided of cilia, even if mucus clearance is allowed by its incessant excretion and gravitational sliding [46]. In vitro studies evidenced that RM-β-CD can enhance the permeation of a lipophilic drug across PC12 cell monolayers, chosen as neuron-like cells, but cannot modify the drug permeation across Caco-2 monolayers, chosen as epithelial-like cells. These data suggest, therefore, that CDs may have different aptitudes in promoting the drug permeation across the respiratory (poor in neuron-like components) or olfactory (rich in neuron-like components) mucosa, with potentially interesting applications in vivo [34].

The nasal formulations obtained in the presence of CDs evidence a high degree of versatility in order to potentiate the local effects of drugs or to promote their absorption at systemic level or in the CNS, as described below.

### 2.2. Nasal Formulations with CDs for the Local Therapeutic Effect

Very recently, nasal powder formulations were designed for a thalidomide complementary anti-epistaxis therapy, necessary when the adverse effects caused by oral treatment of the main therapy require its interruption. Β-CD, SBE-β-CD, and HP-β-CD were used as nasal carriers of these formulations (Table 1). The improved dissolution rate of thalidomide was evidenced for all the formulated powders compared with the raw drug. This behavior suggests that the nasal powders allow a prompt accumulation of thalidomide in nasal environments. Studies of thalidomide transport across excised rabbit nasal mucosa indicated that the nasal powders induce very limited permeation of thalidomide across this type of barrier, suggesting poor systemic absorption upon nasal application. Moreover, β-CD appeared to be able to improve the drug’s accumulation within the tissue, an important aspect for potentially efficient therapeutic effects [47].

### 2.3. Toxicity Studies of CDs

The knowledge of the effects of CDs on nasal mucosa is important when nasal formulations are designed. A study evaluated the in vivo histological effects of β-CD, HP-β-CD, and RM-β-CD on the nasal mucosa of rats. Five minutes of exposure with 1.5% (*w*/*v*) β-CD or 5% and 20% (*w*/*v*) HP-β-CD solutions did not appear toxic for nasal mucosa; on the other hand, the same time of exposure with 20% (*w*/*v*) RM-β-CD solution induced severe damage on the integrity of nasal mucosa. Sixty minutes of exposure with 10% (*w*/*v*) HP-β-CD or RM-β-CD solution did not appear toxic for nasal mucosa, indicating that less than 10% (*w*/*v*) CD solutions can preserve the integrity of the nasal mucosa [48].

RPMI 2650 human nasal septum tumor epithelial cells were used to study the cytotoxicity of α-CD and β-CD by MTT assay. The CDs did not show toxicity at concentrations up to 0.3% (*w*/*v*), whereas 1% (*w*/*v*) solutions induced a significant decrease in the metabolic activity of the cells. According to these data, α-CD and β-CD are suggested to be used in 0.3% (*w*/*v*) concentrations in nasal formulations [49].

Very recently, a toxicity study of new cationic β-CD and their polymers obtained by crosslink with epichlorohydrin was performed in vitro on PC12 and Caco-2 cells, selected to mimic the respiratory and olfactory epithelia of the nasal cavity. The tested compounds evidenced dose- and time-dependent toxicity; the exposure for 60 min of 5 µM quaternary-ammonium-β-CD soluble polymer was recognized as non-toxic [50].

### 2.4. CDs and Modulation of Drug Permeation across Lipophilic Membranes

Several in vitro or ex vivo studies have been performed to evaluate the potential ability of CDs to modulate the drug permeation across nasal mucosae (Table 1). As an example, it has been evidenced that the anti-rhinovirus drug Disoxaril increases its stability and water solubility when complexed with DM-β-CD, which appeared able to increase the drug permeation across excised bovine nasal mucosa [51].

A series of nasal formulations were obtained with micronized melatonin and hydroxypropyl methylcellulose (HPMC), where HP-β-CD or RM-β-CD were added at different concentrations. The permeation of melatonin across EpiAirway™-100 cultures was significantly increased by the presence of CDs at low concentrations (1% (*w*/*v*)), whereas higher concentrations of CDs (up to 10% (*w*/*v*)) induced a significant decrease of the drug permeation [52].

Tacrine nasal formulations were obtained as albumin nanoparticles carrying β-CD, HP-β-CD, or SBE-β-CD. It was evidenced that the CDs were able to modulate the drug loading in the nanoparticles and their mucoadhesiveness. Ex vivo experiments performed on sheep nasal mucosa evidenced the ability of the CDs to increase the drug permeation [53].

A thermally triggered mucoadhesive in situ nasal gel based on poloxamer 407 and carbopol 934 was developed for loratidine, whose solubility was increased by inducing its inclusion complex with β-CD. The formulations containing the loratidine/β-CD complex evidenced higher drug permeation across sheep nasal mucosa than the formulations containing free loratidine [54].

Buspirone hydrochloride microemulsions were designed as nasal formulations, obtained with the drug alone or in combination with chitosan aspartate and HP-β-CD. The buspirone permeation across sheep nasal mucosa was increased by chitosan and optimized by further addition of HP-β-CD in the nasal formulation [55].

As described above, RM-β-CD appears able to enhance the drug permeation across neuron-like monolayers but not across epithelial-like monolayers, suggesting the different aptitude of the CDs in influencing the permeability of respiratory or olfactory mucosae [34].

The role of HP-β-CD as nasal absorption enhancer was evidenced by ex vivo permeation studies on sheep nasal mucosa of in situ gel of paliperidone designed for nasal delivery [56].

Nasal formulations of ribavirin were designed as agglomerates of micronized drugs and spray-dried microparticles containing chitosan or α-CD. Ex vivo transport studies across rabbit nasal mucosa evidenced the inability of chitosan to increase the permeation of ribavirin, differently from α-CD, whose presence in the solid nasal formulation induced a high drug permeation across the biological barrier [57].

The inclusion complex of idebenone with HP-β-CD was evidenced to sensibly increase the water solubility of the drug and its permeation across bovine nasal mucosa, suggesting that idebenone/HP-β-CD complexes are suitable for nasal administration in order to increase the therapeutic performances of idebenone as neuroprotector [58].

The cationic quaternary-ammonium-β-CD monomer and polymer described above for toxicity studies were analyzed with regard to their uptake in Caco-2 cells, selected as epithelial-type cells to mimic the respiratory mucosa. Both the monomer and the polymer appeared internalized in Caco-2 cells and, in particular, the cationic monomer evidenced a higher permeability than HP-β-CD. Therefore, the cationic CDs and their polymers appear as potential excipients for nasal formulations designed to improve the nasal absorption of drugs [50].

Novel nasal formulations of curcumin were designed as curcumin-encapsulated chitosan-coated poly (lactic-*co*-glycolic acid) nanoparticles (CUR-CS-PLGA-NPs) and curcumin/HP-β-CD inclusion complexes. In vitro studies performed on SH-SY5Y cells evidenced higher cellular uptake for curcumin/HP-β-CD inclusion complexes than nanoparticles, reducing the cellular toxicity of curcumin [59].

Inclusion complexes of quercetin with RM-β-CD and HP-β-CD were found to increase the solubility and stability in water of the drug. Moreover, ex vivo permeation studies across rabbit nasal mucosa evidenced the ability of both inclusion complexes to significantly increase the negligible permeability of quercetin across this physiological barrier [60].

Very recently, it was demonstrated that RM-β-CD, included in a thermosensitive nasal in situ gel, is essential to promote clonazepam release and permeation through Caco-2 cells as well as to reduce its cytotoxicity [61].

### 2.5. CDs and Modulation of Systemic Absorption of Nasally Administered Drugs

Several nasal formulations containing CDs were proposed in order to optimize the systemic absorption of drugs (Table 1). For example, a nasal spray solution of midazolam was obtained by solubilizing the drug in water (pH 4.3) in the presence of SBE-β-CD and HPMC. This nasal spray administered to human volunteers obtained an absolute bioavailability of about 70% for midazolam [62].

Nasal formulations of calcitonin were obtained by its dissolution in isotonic phosphate buffer in the absence or the presence of chitosan or DM-β-CD, chosen as absorption enhancers. The nasal administration of the formulation without absorption enhancers (control solution) to rats obtained an absolute bioavailability of 1.2%, whereas the formulation including chitosan induced an absolute bioavailability of 2.4%. The presence of DM-β-CD weakly improved the absolute bioavailability with respect to the control solution, with a value of 1.95%. According to these data, the cationic chitosan appeared as a better systemic absorption enhancer than DM-β-CD for nasally administered calcitonin [63].

Nasal formulations of midazolam obtained as inclusion complexes with RM-β-CD appeared very efficacious for the systemic drug absorption, showing bioavailability values around 90%. The inclusion of chitosan in the formulations allowed to increase the absorption rate of the drug [64].

### 2.6. Nasal Formulations and the Modulation of Central Drug Absorption by CDs

The role of CDs in nasal formulations appears important not only for the systemic absorption of the drugs but also for the brain targeting of neuroactive agents (Table 1). About ten years ago, an interesting study evidenced that the pituitary adenylate cyclase-activating polypeptide (PACAP), a potent neuro-protectant, when nasally administered, was able to reach mainly the occipital cortex and striatum of the brain. The nasal formulation was obtained with lactated Ringer’s solution (LR) and bovine serum albumin. The addition of appropriate CD in the formulation allowed the selection of specific brain regions for the brain targeting of PACAP. In particular, the uptake by the olfactory bulb was increased by α-CD, which, instead, decreased the uptake by the occipital cortex and striatum; the uptake by the hypothalamus and the occipital cortex was increased by β-CD. The uptake by the thalamus was increased by HP-β-CD, which, instead, decreased the uptake by the striatum. These data suggest that neuroactive polypeptides may be targeted in specific brain regions by choosing the appropriate CD in nasal formulations [65]. Similarly, 17β-estradiol (E2), proposed to improve spatial learning and memory, appears influenced by CD in targeting specific brain regions upon nasal administration [66].

A nasal formulation for carnosic acid was designed to target this drug in the brain and enhance the endogenous levels of neurotrophins with consequent prevention and treatment of neurodegenerative disorders. Carnosic acid’s solubility in water was enhanced in the presence of HP-β-CD. Aqueous solutions of this complex were administered to rats in the absence or the presence of chitosan by the subcutaneous or nasal way. The nasal formulations characterized by the presence of chitosan appeared to be able to optimize the production of neurotrophins in the brain of rats [67].

Another study on a neuroactive peptide, the glucagon-like peptide-2 (GLP-2), evidenced that a nasal formulation containing polyoxyethylene lauryl ether and β-CD induced its brain targeting of GLP-2 and antidepressant-like effects [68].

An innovative nanoparticulate nasal formulation of dopamine was obtained with glycol-chitosan and SBE-β-CD, which was used as the crosslinking agent and to enhance the drug stability. Acute administration of this formulation in the nostril of the rats allowed to target their olfactory bulb but not their striatum, which was instead targeted following repeated intranasal administrations [69].

The above-described nasal formulation of ribavirin, constituted by agglomerates of micronized drug and α-CD spray-dried microparticles, was able to increase, upon nasal administration, the drug accumulation in several brain compartments (olfactory bulb, basal ganglia, hippocampus; anterior cortex, posterior cortex, cerebellum) in comparison with the micronized ribavirin without excipients. Moreover, the nasal administration of the formulation containing α-CD was able to increase the ribavirin olfactory bulb/plasma ratio by about five times with respect to the intravenous administration of ribavirin [57].

Benzodiazepines intranasally administered do not appear to be transported from nose to the brain; after nasal administration, these drugs reach the CNS quickly by the systemic pathway. This behavior obtains efficacious therapeutic effects against acute repetitive seizures by nasal administration of benzodiazepines [70]. However, it has been evidenced that the nasally administered neuroactive steroid allopregnanolone is faster in producing the same central effects of nasally administered benzodiazepines, with the further advantage of not producing peripheral unwanted effects [71]. A nasal formulation for allopregnanolone was obtained by its dissolution in saline solution in the presence of SBE-β-CD, which, upon administration to mice, induced a direct nose-to-brain delivery of the drug, whose higher brain levels were detected in the olfactory bulbs. Interestingly, the olfactory bulb axons project directly to regions of the brain involved with the seizure protection of antiepileptic drugs [71].

The water solubility of berberine, proposed as an antidepressant, was increased by preparing an inclusion complex with HP-β-CD. A nasal formulation was designed by loading the inclusion complex in a thermoresponsive hydrogel obtained with poloxamers P407 and P188. Upon nasal administration to rats, this formulation obtained a relative bioavailability of berberine in the hippocampus 110 times higher than that obtained by the oral administration of the drug/HP-β-CD inclusion complex. Nasal administration improved, moreover, the antidepressant activity of berberine [72].

A temperature/ion-sensitive in situ hydrogel was designed as a nasal formulation for timosaponin BII, an efficient anti-Alzheimer disease agent. The gel was obtained with deacetylated gellan gum and poloxamer 407 in the presence of HP-β-CD. This formulation was intranasally administered to mice for 38 days, inducing therapeutic efficiency against an Alzheimer disease model and high brain targeting of timosaponin BII [73].

Disulfiram, proposed against glioblastoma, was included in an inclusion complex with HP-β-CD. The disulfiram/HP-β-CD complex combined with copper ions was nasally administered, showing efficient therapeutic effects against intracranial glioma-bearing male rats. A strong brain-targeting of nose-to-brain drug delivery was confirmed by fluorescence signal of the fluorescent dye Cy5.5/HP-β-CD [74].

### 2.7. Nasal Formulations and CDs Modulation of Systemic and Central Absorption of Neuroactive Agents

Several studies were performed to evaluate the potential existence of a direct nose-to-brain transport of nasally administered neuroactive agents (Table 1). The use of CDs in nasal formulations has often allowed this pathway to be enhanced. As an example, an inclusion complex of estradiol with RM-β-CD was designed for its nasal and intravenous administration to rats. The AUC ratio of estradiol between CSF and plasma related to intravenous administration increased about three times (from about 0.5 to 1.5) when the drug was nasally administered, suggesting a direct transport of estradiol from the nose to the CSF via olfactory neurons [75].

A new formulation of the precursor of dopamine, L-dopa, was prepared as an inclusion complex with HP-β-CD in maleic acid for nasal administration. Concerning oral administration, the nasal way allowed to increase the drug absorption in both the bloodstream and the brain of rats [76].

The buspirone hydrochloride microemulsion, described above, when nasally administered, was able to increase approximately eight-fold the AUC ratio of the drug between the brain and plasma concerning the nasal or intravenous administration of a buspirone solution. This result was optimized by the concomitant presence of HP-β-CD and chitosan in the formulation [55].

Solid nasal formulations were obtained for deferoxamine, an anti-ischemic agent, by spray-dying in the presence of chitosan chloride, or RM-β-CD. The nasal administration to rats of these formulations allowed to obtain deferoxamine absolute bioavailability values of 6% and 15% in the presence of chitosan and HP-β-CD, respectively. Moreover, a direct nose to CSF pathway was identified for deferoxamine following the nasal administration of the solid formulations. The drug concentrations in CSF obtained in the presence of HP-β-CD were about four times higher than those obtained in the presence of chitosan [34].

Very recently, it has been demonstrated that the nasal administration to rats of HP-β-CD microspheres, based on mucoadhesive polymers (sodium alginate and chitosan), protects against the neurotoxicity produced by β-amyloid plaques in the brain. A synergic role of HP-β-CD and chitosan was identified in neuroprotection [77].

Scutellarin, proposed as an anti-ischemic agent, was encapsulated into nanoparticles based on chitosan and obtained in the presence of HP-β-CD by ionic crosslinking with sodium tripolyphosphate. Compared with the nasal administration of a scutellarin solution to mice, the nanoparticles did not induce absorption advantages of the drug in the bloodstream, whereas in the brain, they induced the doubling of drug uptake. Moreover, the drug absorption in both plasma and brain of mice appeared advantageous following the nasal administration of the nanoparticles, concerning their oral administration [78].

Microspheres based on both RM-β-CD and chitosan were prepared as nasal formulations of a polar anti-ischemic agent by spray-drying. The solid microspheres were nasally administered to rats to analyze their ability to induce systemic or CSF delivery of the drug. In particular, the microspheres containing RM-β-CD and chitosan at a 50:50 weight ratio were compared with microspheres containing a single excipient. A direct nose-to CSF delivery of the drug was evidenced by the three formulations. The increase of RM-β-CD amounts in the microspheres enable an increase in the drug uptake in both the bloodstream and the CSF, optimizing the drug distribution between the CSF and the bloodstream [37].

An interesting nasal formulation of butylidenephthalide, an efficient anticancer drug, was obtained by encapsulating in liposomes its hydrophilic inclusion complex with HP-β-CD, designed to increase the drug-loading capacity [79]. The nasal administration of this drug formation, produced in the brains of mice, amounts to being ten times higher than those obtained by oral administration. The nasal formulation appeared efficient against temozolomide-resistant glioblastoma [79].

A thermosensitive in situ gel for nasal delivery was designed for donepezil, a neuroactive agent proposed for the therapy of Alzheimer’s disease. The formulation was obtained with poloxamers 407 and 188, in the presence of HP-β-CD, chosen as a permeation enhancer. The nasal administration of this formulation was more efficient to induce drug absorption in both the plasma and the brain of rats compared with an intragastric administration of the drug solution. The drug targeting efficiency related to the nasal administration of in situ gel formulation was higher than 150%, suggesting its aptitude to deliver in the brain drugs amounts higher than in the plasma [80].

The novel nasal formulations of curcumin above described, CUR-CS-PLGA-NPs and curcumin/HP-β-CD inclusion complexes, were able to induce the drug absorption in the plasma and the brain following nasal administration. In both compartments, the drug absorption appeared more efficient with curcumin/HP-β-CD in comparison with CUR-CS-PLGA-NPs [59].

Geraniol is a plant metabolite proposed for the therapy of Parkinson’s disease. Its delivery in the CNS via nose-to-brain transport caused strong nasal mucosae damages when an emulsified formulation was used [40]. Considering these aspects, β-CD and HP-β-CD were proposed as geraniol carriers for the geraniol delivery in the brain after nasal administration. The water solution of geraniol/HP-β-CD complex and the water suspension of geraniol/β-CD were nasally administered to rats, obtaining a direct nose-to-CSF targeting of geraniol but not its absorption in the bloodstream. Both formulations preserved the integrity of the nasal mucosa. Singularly, the nasal administration of the geraniol/β-CD suspension allowed to obtain geraniol amounts in CSF of rats two orders of magnitude higher than those obtained by using the geraniol/HP-β-CD solution. This result was attributed to the potential aptitude of geraniol/β-CD complex to form nanoparticulate aggregates able to reduce the interaction of geraniol with mucus, allowing a direct deposition of the drug on the olfactory mucosa [41].

### 2.8. CDs as Carriers of Nasal Vaccines

β-CD conjugated with polyethyleneimine was also studied as an intranasal delivery system able to prolong the residence and increase the transport of mRNA vaccines in the nasal mucosa [81]. The cationic cyclodextrin-polyethyleneimine polymer was able to interact with negatively charged mRNA, forming a nanocomplex [81]. These systems showed higher in vitro transfection efficiency and strong in vivo abilities to migrate to the lymph nodes and stimulate dendritic cell maturation, with low local and systemic toxicity [82].

## 3. Cyclodextrins as Actives

Due to the ability to interact with membrane cholesterol, CDs show biological activities useful for their applications as active ingredients. In particular, as a cholesterol-sequestrating agent, CDs have antiviral, antiparasitic, and anti-atherosclerotic activities as well as beneficial effects in some neurodegenerative disorders (Niemann-Pick type C, Alzheimer’s, Parkinson’s, and Huntington’s diseases) [83,84]. Additionally, CDs can directly bind β-amyloid peptides (Aβ), inhibiting amyloid fibril formation and thus decreasing the neurotoxic effect in vitro and in vivo [85,86,87].

On 26 April 2013, HP-β-CD received the orphan designation (EU/3/13/1124) by the EMA for the treatment of Niemann-Pick disease type C, and it is already the object of a Phase 3 study in Pediatric and Adult Patients both in the US and the EU (ClinicalTrials.gov Identifier: NCT04860960). In these studies, HP-β-CD is administered by intrathecal or intravenous infusion due to its pharmacokinetics [88]. The volume of distribution (VD) after intravenous injection is about 0.2 L/kg in humans, and t½ is about 1.6–1.7 h; it is excreted within 6 h and does not cross the BBB significantly [89]. For that, very high doses of HP-β-CD have been parenterally administered, resulting in more side effects [88].

For the use of CDs as active ingredients for the treatment of brain diseases, formulation strategies are necessary. With this aim, Gavini and co-workers prepared nasal microspheres based on chitosan and/or alginate for direct nose-to-brain transport of HP-β-CD and β-CD [86]. Both polymers are mucoadhesive, whereas chitosan is also a penetration enhancer widely used in nasal formulations [31,32,34,35,90,91]. Eight batches of spray-dried microspheres were produced and characterized. The microspheres had volume-surface diameters of 2.1–3.7 μm, suitable for nasal administration, almost spherical shapes with invaginations and smooth surfaces, and good water uptake ability. The formulations controlled the in vitro CD’s release. The alginate formulation showed the best in vitro performance [86]. On the contrary, after in vivo nasal administration for seven consecutive days to Alzheimer’s disease rat model, chitosan-based microspheres were more efficient in reducing the oxidative stress and apoptotic parameters [77]. The results obtained demonstrated that HP-β-CD can reach the brain after nasal administration, particularly the hippocampus, where it provides a protective action against Aβ-induced excitotoxicity [77,92].

Rassu and collaborators recently found that two CD polymers, the soluble amino-β-CD polymer and the soluble β-CD polymer, showed the ability to reduce oxidative damage in epithelial and neuron-like cell lines, opening up new possibilities of use of these derivatives as potential drugs [50].

### CDs for the Control of Viral Infections

In 2016, Kusakabe et al. suggested HP-β-CD as an adjuvant for seasonal and pandemic influenza vaccines. After intranasal administration, the influenza vaccine containing HP-β-CD induced the secretion of specific IgA and IgG antibodies both in the nasal mucosa and in the blood, providing higher and complete protection against infection than the vaccine without HP-β-CD [93]. The mechanism action of HP-β-CD resulted in the induction of the temporary release of IL-33 cytokines from alveolar epithelial cells in the lung; this effect was not observed after subcutaneous injection either after intranasal administration of other vaccine adjuvants [94]. Furthermore, HP-β-CD is safer than aluminum salt because it induces a low production of IgE antibodies [93]. In Japan, a Phase 1 clinical study of the HP-β-CD-adjuvanted influenza split vaccine is underway (NIPH Clinical Trials UMIN ID: UMIN000028530).

CDs were also proposed for the control of the COVID-19 pandemic. The nasal applications of mouth rinses containing β-CD combined with Citrox could lower the viral load in the nasopharynx, useful for preventing of SARS-CoV-2 infection [95].

## 4. Experimental Models for Studying Nasal Drug Delivery

### 4.1. Cell Lines as In Vitro Models to Investigate Drug Permeation across the Nasal Mucosa

Unfortunately, most of the in vitro studies on nasal drug delivery have reported results obtained in surrogate models, as cell lines expressing physiological features of the nasal epithelium have been missing or not properly cultured for a long time. Due to this lack, nasal epithelial cells have been replaced by human bronchial epithelial cell lines, such as Calu-3 and 16HBE14o-, by many research groups despite the known morpho-functional differences of the two cell types [96].

However, animal models have been significantly replaced with established human nasal epithelial cell culture in vitro systems based on primary culture techniques. Actually, primary cell cultures are known to be very suitable systems for the study of nasal epithelial permeability and drug transport, and in particular human-derived nasal cells may confer more clinical relevance to these studies. However, the difficulty in obtaining reliable sources of human tissue is the main factor impairing an extensive use of primary cell culture models of the human nasal epithelium [97].

Moreover, because of the different origins of human subjects, these primary cell culture models suffer from a general lack of reproducibility in the monolayer formation. Furthermore, the short lifespan of primary cells also represents a drawback [96]. The morphology, barrier formation, permeation properties, and drug transporter expression of human nasal epithelial cells are known to be affected by culture conditions, and these parameters have to be taken into account when establishing and validating cell models in vitro. Therefore, an exhaustive characterization of a nasal epithelial cell model and its permeability properties is necessary to achieve a standardized model applicable for the design of therapeutic aerosols and drug transport studies [98].

Establishing adequate cell lines can increase their proliferation and extend their time in culture while saving costs and providing reproducible results [97]. Several cell lines have been developed as nasal in vitro models to study the transport and metabolism of drugs. The immortal nasal cell lines on which drug transport has been analyzed are essentially the RPMI 2650, BT, and NAS2BL cells. The latter two are of animal origin and are derived from bovine turbinate (BT) and rat nasal squamous epithelium carcinoma (NAS2BL), respectively [96]. The unique immortalized human nasal cell line commercially available is RPMI 2650 (ATCC CCL-30 or ECACC 88031602). The RPMI cell line was derived from an anaplastic squamous cell carcinoma of the human nasal septum and resembles normal human nasal epithelium cells to karyotype, its cytokeratin polypeptide pattern, and secretion of cell surface mucins [99]. The cost and reproducibility of results are regarded as advantageous aspects of this in vitro nasal model, as the RPMI 2650 cell line is considered to be higher performing than human primary cell cultures and the resected human tissue model [99]. In addition, although several studies have reported the ineffective differentiation that leads only to multilayered non-ciliated cells lacking polarization as the main drawback of RPMI 2650 cells, it has to be pointed out that in these studies, RPMI 2650 cells were not cultured in the air–liquid interface (ALI), a condition now known to be instrumental for the differentiation of a polarized monolayer of respiratory and nasal epithelial cells. Actually, the ALI is a cell culture condition where a growth medium is added only to the basolateral compartment of the filter insert-based 3D-culture system, whilst the apical domain of the cell monolayer is in direct contact with the air. In the ALI condition, RPMI 2650 cells significantly improved their differentiation and expressed better bioelectrical properties due to increased aerobic respiration compared to that observed when they were totally immersed in the medium [96].

Furthermore, the proliferation and differentiation of RPMI 2650 cells, as well as of many other cell types, also strictly depend on cell scaffold type, seeding density, the composition of the proteins of the extracellular matrix such as collagen, fibronectin, glycosaminoglycans, and glycoproteins, among which type 1 collagen was found the best component to promote RPMI 2650 cell differentiation [96]. In summary, RPMI 2650 barrier properties are greatly dependent on cell culture conditions, such as collagen-coated inserts and the physiological ALI, upregulating the expression of both tight junction proteins and drug transporters, while the growth medium and type of insert have a minor influence in the building a nasal barrier. The ALI is the key culture condition to be considered when using the RPMI 2650 cell line as an in vitro model resembling nasal mucosa for drug permeability studies and screening of nasal drug candidates [96,100].

To date, no relevant publications employing CDs on drug permeation and transport across RPMI 2650 cell monolayers are known, probably due to the drawbacks in achieving a tight monolayer by this cell line mentioned above. However, it is desirable that adjuvanted by the experimental measures suggested, this cellular model of nasal epithelium can currently be considered suitable for transport studies supported by CDs. Only one study on cytotoxicity by α-CD and β-CD in RPMI 2650 cells has been performed, concluding that α-CD and β-CD and methylcellulose, as permeability enhancers in nasal formulations, have to be used in 0.3% (*w*/*v*) concentrations to avoid toxicity measured by (3-(4,5-dimethylthiazol-2-yl)-2,5-diphenyltetrazolium bromide (MTT) assay and impedance measurements [49]. A very recent study by Rassu and colleagues [50] has demonstrated the low cytotoxicity and good cell uptake of cationic β-CD using a rat pheochromocytoma PC12 cell line and a rat intestinal Caco-2 cell line as models for the neuronal and epithelial cells, respectively [50]. This concomitance of cell models was chosen based on the organization of nasal epithelium, according to which, drugs can reach the systemic circulation or the endings of the trigeminal nerve and then reach the brain through the respiratory component while crossing the olfactory epithelium. Through the olfactory neurons or the olfactory epithelial cells, drugs can directly access the CSF or the brain. We would suggest that a similar experimental approach could also be set up and/or implemented using a conditionally immortalized cell line derived from the olfactory sensory neuron (OSN) lineage, termed Odora cells [101], although they are much less easy to be retrieved. Undifferentiated Odora cells have a phenotype similar to OSN progenitors, i.e., the globose basal cells, but differentiated Odora cells more closely resemble OSNs and express neuronal and olfactory markers, including components of the olfactory signal transduction pathway. A coculture between Odora cells and RPMI 2650 cells could be proposed as an interesting in vitro model mimicking both the respiratory and the olfactory components of the nasal epithelium and, therefore, fully applicable to study drug absorption enhancers as CDs.

### 4.2. Combining Intranasal Administration with Plasma and Cerebrospinal Fluid Sample Collections: Useful Strategies to Evaluate Nose-to-Brain Drug Delivery in Preclinical Studies

To evaluate the nose-to-brain drug delivery in preclinical studies, including those aimed at establishing the potential ability of CDs to modulate the drug permeation across nasal mucosa, the choice of a good animal model and experimental protocol is crucial. The use of a species with a close analogy to the human nose-to-brain transport route represents the best option. In this context, non-human primates (NHPs), which share structural and functional features with human brains, surely would be the optimal choice. While this similarity represents an undoubted scientific advantage, the use of NHPs is limited by several disadvantages such as the experimental costs, the difficulty to apply experimental procedures on such complex animals, and some relevant ethical problems. On the contrary, rats are easy to handle, relatively inexpensive, and being larger than mice allows for the possibility to sequentially collect plasma and cerebral fluid samples after the tested substances intranasal administrations.

As a consequence, most studies evaluating the impact of CDs on nose-to-brain drug delivery were carried out in rats [41,57,69,74]. Usually, intranasal drug administration is performed on anaesthetized rats either for ethical reasons or to avoid (i) damaging the nasal mucosa with the drug delivery device; and (ii) losing part of the administered dose. To intranasally administer the required drug amount, the anesthetized rat is placed on its back or in the right lateral recumbency [39,57], and the drug is administered by introducing into one or each nostril the required microliters of the formulation under investigation. To avoid incidental damage of nasal mucosa and to be sure to precisely administer the required volume, the use of a semiautomatic pipet attached to a short polyethylene tubing inserted approximately 0.5–0.7 cm into each nostril is suggested [39,41]. After that, biological fluid sample collection can also be performed on the awake, freely moving animal.

To assess the extent of nose-to-brain drug delivery following the described above administration procedure, it becomes necessary to quantify the drug amount into the brain. CSF sample withdrawal using the cisternal puncture method described by van den Berg et al. [102], which requires the stereotaxic placement of a single needle stick connected to a CSF collection tubing into the rat cisterna magna, is, at least in our opinion, an optimal procedure [33]. This methodology is, in fact, easy to perform and presents several advantages over previously reported CSF sampling methods (e.g., microdialysis or the cisternal cannulation methods), such as low-risk of local infection, low-risk of blood contamination into the collected CSF samples, and a faster sampling speed, which results in an accurate CSF sampling [102]. Importantly, this procedure permits the collection of serial (40–50 μL) CSF samples, thus allowing to evaluate the pharmacokinetics of the intranasally administered drug in the CNS. The possibility to couple CSF sampling with sequential blood sample collection represents a further advantage of this experimental procedure, thus satisfying a fundamental requirement for an animal model to be used to evaluate nose-to-brain drug delivery in preclinical studies [102]. In addition to the procedures mentioned above, in several studies, the brain drug delivery has been established based on the finding of the drug in extracts from the brain removed from the animal after its sacrifice. It is worth noting that this method can be considered reliable only following the complete removal of the blood from the brain (i.e., by intracardiac physiological solution perfusion) since the drug present into cerebral circulation can contaminate the samples.

Furthermore, this approach only allows researchers to obtain a single drug concentration value in animals. Therefore, this procedure is rather complicated, needs in vivo intracardiac saline perfusion, and requires the sacrifice of a high number of animals in case of the necessity to perform pharmacokinetic studies. Thus, also based on of the three R’s of Russell and Burch: Replacement, Reduction and Refinement [103], requiring that the experimental set-up should have the least possible degree of discomfort for the animals, and should necessitate the lowest animal number, the rat cisternal puncture method appears to be the most suitable CSF sampling procedure to evaluate the nose-to-brain drug delivery in preclinical studies.

## 5. Conclusions

CDs are oligosaccharides usually used as complexing agents to improve the physicochemical properties of drugs and in recent decades have been investigated for new applications. In nasal drug delivery, CDs can be useful both as pharmaceutical excipients (solubilizer and absorption promoter) and actives (antiviral, antiparasitic, anti-atherosclerotic, and neuroprotective), considering their non-toxicity from clinical data. Studies performed by several Italian research groups significantly contributed to the development of these topics. The use of CDs in nasal formulations allowed researchers to obtain versatile drug delivery systems intended for local and systemic effects, as well as to achieve therapeutic drug concentrations in CNS. In general, CDs contribute to increase the drug solubility in water and modulate their permeability across the nasal mucosa. In terms of the systemic effects, the presence of CDs in nasal formulations has been demonstrated to increase the bioavailability of sedatives and drugs against hypercalcemia. As far as the central effects are concerned, different types of CDs in nasal formulations appeared to induce an increase in drug uptake in different regions of the brain. In vitro and in vivo models are currently suitable to analyze the effects produced by the presence of CDs in nasal formulations. Interesting perspectives, however, appear to simulate the olfactory mucosa in vitro. The rat emerges as one of the most appropriate animal models for in vivo studies because they allow conjugating the low costs with the opportunity to collect plasma and cerebral fluid samples during the time sequentially. Therefore, CDs are versatile pharmaceutical materials with potential biological activities, and their nasal application could represent an interesting and fruitful research field in the coming years.

## Figures and Tables

**Table 1:** Formulations containing CDs designed for nasal administration as described in Section 2.

CD Type	Drug	Formulation Type	Other Excipients	Experimental Procedures	Aim	Ref.
β-CD, HP-β-CD, SBE-β-CD	Thalidomide (anti-epistaxis therapy)	Nasal powder	Lecithin	Permeation across ex vivo rabbit nasal mucosa	Local action	[47]
HP-β-CD; RM-β-CD, β-CD	--	Sodium phosphate solutions of CDs	--	In vivo administration to rats and histological analysis of nasal mucosa	Integrity analysis of mucosa	[48]
α-CD, β-CD	--	Solutions in Eagle’s minimal essential medium	β-D-mannitol, sodium hyaluronate, polyvinyl alcohol, methyl-cellulose (as an alternative to CDs)	Toxicity analysis on RPMI 2650 human nasal septum tumor epithelial cells	Toxicity analysis of several excipients	[49]
Positively charged β-CDs and their polymers	--	Hank’s salt solutions	--	Toxicity and internalization analysis on PC12 and Caco-2 cells	Model of nasal absorption	[50]
DM-β-CD	Disoxaril (WIN 51711) (Antiviral)	Inclusion complexes in PBS	HPMC	Permeation across ex vivo bovine nasal mucosa	In vitro and in vivo studies on the nasal mucosa	[51]
HP-β-CD; RM-β-CD	Melatonin (MT) (sleep disorders)	Gel formulations with CDs and micronized MT	HPMC	Permeation on EpiAirway™-100 cells	Model of nasal absorption	[52]
SBE-β-CD; HP-β-CD	Tacrine (Alzheimer’s disease)	Albumin NP with CDs	Albumin	Permeation across ex vivo sheep nasal mucosa	Model of nasal absorption	[53]
β-CD	Loratidine (antihistaminic)	Mucoadhesive nasal gel	Poloxamer 407, carbopol 934P	Permeation across ex vivo sheep nasal mucosa	Model of nasal absorption	[54]
HP-β-CD	Buspirone hydrochloride (BH) (anxiolytic)	Microemulsion	Chitosan aspartate	Permeation across ex vivo sheep nasal mucosa; in vivo BH administration to rats and quantification in plasma and brain	Study of CD effect on BH brain uptake following nasal administration	[55]
RM-β-CD	Deferoxamine (DF) (neuroprotective)	Spray-dried microparticles	Chitosan chloride (as an alternative)	Permeation studies across PC 12 and Caco-2 cells; in vivo DF administration to rats and quantification in plasma and CSF	Study of CD effect on DF brain uptake following nasal administration	[34]
HP-β-CD	Paliperidone (PLPD) (treatment of schizophrenia)	Nasal in situ gel	Carbopol 934, HPMC	Permeation across ex vivo sheep nasal mucosa; mucoadhesive studies	Model of nasal absorption	[56]
α-CD	Ribavirin (RBV) (antiviral)	Micronized RBV and microspheres	Lecithin and mannitol, chitosan	Permeation across ex vivo rabbit nasal mucosa; in vivo RBV administration to rats and quantification in plasma and brain	Study of formulative effects on RBV brain uptake following nasal administration	[57]
HP-β-CD	Idebenone (IDE) (antioxidant)	IDE/HP-β-CD inclusion complex	--	IDE protection studies in human glioblastoma astrocytoma (U373) cells; permeation across ex vivo bovine nasal mucosa	Model of nasal absorption and drug protection in glioblastoma	[58]
HP-β-CD	Curcumin (CUR) (Alzheimer’s disease)	CUR/HP-β-CD inclusion complexes	PLGA nanoparticles chitosan-coated (as an alternative)	Uptake studies on SH-SY5Y cells; in vivo CUR nasal administration to mice and quantification in plasma and brain	In vitro and in vivo comparison between inclusion complexes and nanoparticles	[59]
RM−β-CD	Quercetin (Que) (Alzheimer’s disease)	Que/RM-β-CD inclusion complex	--	Permeation across ex vivo rabbit nasal mucosa	Model of nasal absorption	[60]
RM-β-CD	Clonazepam (CLZ) (antiseizure)	CLZ/RM-β-CD inclusion complex in thermosensitive, in-situ gel	Poloxamer 407, sodium hyaluronate and chitosan glutamate	Cytotoxicity and transport studies on Caco-2 cells	Study of formulative effects on CLZ systemic delivery	[61]
SBE-β-CD	Midazolam (sedative)	Aqueous nasal spray	HPMC	In vivo pharmacokinetic studies on human volunteers	Study of systemic absorption from nasal way	[62]
DM-β-CD	Salmon Calcitonin (hypercalcemia)	Solutions	Chitosan (as an alternative)	In vivo bioavailability studies on rats	Study of systemic absorption from nasal way	[63]
RM-β-CD	Midazolam (sedative)	Aqueous solutions	Chitosan	In vivo bioavailability studies on human volunteers	Study of systemic absorption from nasal way	[64]
β-CD, α-CD, HP-β-CD	PACAP ^1^ (neurotrophic and neuroprotectant)	Ringer’s solution with BSA	BSA	In vivo uptake studies in brain regions after nasal administration to mice	To study the influence of CDs on PACAP uptake in different brain regions	[65]
β-CD, HP-β-CD, RM-β-CD, γ-CD	17β-estradiol (E2) (spatial learning and memory)	Saline	NaCl 0.9%	In vivo uptake studies in brain regions after nasal administration to rats	To study the influence of CDs on PACAP uptake in different brain regions	[66]
HP-β-CD	Carnosic acid (neuroprotective)	Aqueous solutions	Chitosan	In vivo studies of brain neurotrophin production after nasal administration to rats	To study the influence of CD on brain uptake of carnosic acid	[67]
β-CD	Glucagon-like peptide2 (GLP-2) (antidepressant)	Phosphate buffer solution	Polyoxyethylene; lauryl ether	In vivo uptake studies in brain regions after nasal administration to rats	To study the influence of CD on GLP-2 uptake in different brain regions	[68]
SBE-β-CD	Dopamine (Parkinson’s disease)	NPs of chitosan crosslinked by SBE-β-CD	Chitosan	In vivo uptake studies in brain regions after nasal administration to rats	Uptake studies in different brain regions of NPs	[69]
SBE-β-CD	Allopregnanolone (antiseizure)	Saline	NaCl 0.9%	In vivo uptake studies in brain regions after nasal administration to mice	Allopregnanolone uptake studies in different brain regions	[71]
HP-β-CD	Berberine (antidepressant)	Thermoresponsive hydrogel	Poloxamers P407 and P188, benzalkonium chloride	In vivo bioavailability studies of berberine in rat hippocampus after oral and nasal administration	To study the influence of nasal formulation on berberine brain uptake	[72]
HP-β-CD	Timosaponin BII (Alzheimer’s disease)	Temperature/ion-sensitive in situ hydrogel	Deacetylated gellan gum; poloxamer	Nasal administration to mice for 38 days; analysis of spatial memory and spontaneous behavior; in vivo fluorescence imaging	Formulation study for local prevention of Alzheimer’s disease	[73]
HP-β-CD	Disulfiram (DSF) (anti-cancer)	DSF/HP-β-CD inclusion complex	--	Nasal administration with Cu; fluorescence and glioma inhibition studies	Evaluation of the distribution and therapeutic effects of the inclusion complex	[74]
RM-β-CD	Estradiol (ES) (menopausal symptoms)	ES/RM-β-CD inclusion complex in saline	NaCl 0.9%	ES pharmacokinetic studies after intravenous or intranasal nasal administration to rats	Comparison of ES distribution between plasma and CSF	[75]
HP-β-CD	L-dopa (Parkinson’s disease)	L-dopa/HP-β-CD inclusion complex in maleic acid aqueous solution	Carbidopa	Pharmacokinetic studies after oral or nasal administration to rats	Comparison of L-dopa distribution between bloodstream and brain	[76]
HP-β-CD	HP-β-CD (neuroprotective)	Spray-dried microparticles	Chitosan, alginate	Neuroprotection studies against amyloid plaques after nasal administration to rats	Neuroprotective studies on brain synaptosomes	[77]
HP-β-CD	Scutellarin (SC) (antiischemic)	HP-β-CD –chitosan nanoparticles	Chitosan, sodium tripolyphosphate	Pharmacokinetic studies on plasma and brain after oral and intranasal administration to mice	Comparison of SC distribution after oral or intranasal administrations	[78]
RM−β-CD	N^6^-cyclopentyl-adenosine (CPA) (antiischemic)	Spray-dried microparticles	Chitosan	Pharmacokinetic studies on bloodstream and CSF after nasal administration to rats	Comparison of CPA distribution in dependence on Me−β-CD and chitosan in MPs	[37]
HP-β-CD	Butylidenephthalide (BP) (anti-cancer)	BP/HP-β-CD inclusion complex encapsulated in liposomes	1,2-dimyristoyl-sn-glycero-3-phospho-choline; cholesterol	BP quantification in the brain of mice after oral and nasal administration	Comparison of antitumor effects between oral and nasal administrations	[79]
HP-β-CD	Donepezil hydrochloride (Alzheimer’s disease)	Thermosensitive in situ gel	Poloxamers 407 and 188, ethylparaben	Pharmacokinetic studies on plasma and brain after intra-gastric and nasal administrations to rats	Comparison of donepezil distribution after intra-gastric and nasal administrations	[80]
β-CD, HP-β-CD	Geraniol (GER) (Parkinson’s disease)	Inclusion GER/β-CD and GER/HP-β-CD complexes in ultrapure water	--	Pharmacokinetic studies on bloodstream and CSF after nasal administration to rats	Comparison of GER distribution after nasal administration of inclusion complexes	[41]
β-CD	mRNA vaccine	Nanoparticles	Polyethyleneimine (PEI600 or PEI2k)	In vivo studies to evaluate the efficacy as an intranasal mRNA vaccine	Evaluation of local and systemic immune responses	[81,82]

^1^ PACAP: Pituitary adenylate cyclase activating polypeptide. Other abbreviations: BSA: Bovine serum albumin; CSF: cerebrospinal fluid; NPs: nanoparticles; MPs: microparticles. CDs abbreviations: α-CD: α-cyclodextrin; β-CD: β-cyclodextrin; DM-β-CD: Dimethyl-β-cyclodextrin; RM-β-CD: Randomly methylated-β-cyclodextrin; HP-β-CD: Hydroxypropyl-β-cyclodextrin; SBE-β-CD: Sulfobutylether-β-cyclodextrin; γ-CD: γ-cyclodextrin.
